# Poor-Law Infirmaries in War-Time: How Difficulties Have Been Surmounted

**Published:** 1915-10-23

**Authors:** 


### POOR-LAW INFIRMARIES IN WAR-TIME.

### How Difficulties have been Surmounted.

During the last few months the War Office has taken over in their entirety a number of Poor-Law institutions throughout the kingdom for the reception of wounded and invalid soldiers. In London, for instance, the workhouses and infirmaries of the City of London, Fulham, Hampstead, Lewisham, Mile End, and Brentford Guardians, the infirmary of the Bethnal Green Guardians, and the Homes for the Aged at Tooting of the Wandsworth Union have been utilised in this way. In nearly all the large provincial towns one or more of the Poor-Law institutions have been acquired. In other towns where it was impossible to hand over the whole of an institution to the War Office without serious hardship to the sick poor, one or more blocks of wards have been emptied of their usual inmates and placed at the disposal of the War Office. In the latter case it has been usual for the War Office to pay the Guardians so much per day for each soldier sent, and the whole of the arrangements necessary have been left in the hands of the Guardians. In some cases the medical staff has received extra remuneration for the additional work thus thrown upon them, in others no payment has been made, and this has been felt to be a grievance, although from patriotic motives no protest and no application for an increased salary have been made.

### Financial Adjustments.

As regards those institutions which have been taken over as a whole, the rule has been for the War Office to take over with the buildings the Guardians' staff wherever it was possible to utilise their services. Members of the staff for whom no place could be found in the war hospital have been transferred to other work under their own board or found employment under other boards. The Local Government Board at the outset laid down the rule that no Board of Guardians should make a profit or a loss out of the financial adjustments necessary, and that no Poor-Law official should suffer either in salary or in superannuation prospects from the changes. This rule has been loyally carried out. The staff of the new war hospital receive either their Poor-Law salary or the salary attached to their office in a military hospital, whichever is the larger. In the case of the assistant medical officers and the ward sisters and staff nurses this has meant an increase in their pay. The medical officers receive 24s. per day, the sisters £50, rising to £65, per annum, with an annual allowance of £8 for uniform, and the staff nurses £40, rising to £45, with a similar uniform allowance. Commissions have been given to the medical staff—the medical superintendents in London being given the rank of major, outside London that of lieutenant-colonel, the assistant medical officers that of lieutenant. The matrons are paid their Poor-Law salaries, with the addition of charge pay, which varies according to the number of beds in the hospital. Both medical and nursing staffs have been considerably increased. The medical staff consists of the officer in charge—in nearly all cases the former medical superintendent—a registrar, three or four commissioned medical men who act as orderly medical officers, and a number of half-time civil practitioners. In addition, certain special appointments have been made, including a radiographer, an ophthalmic surgeon, and other specialists.

### A Question of Nurse Training.

The nursing is carried out by sisters and staff nurses, who must have had three years' training in a recognised school, by Voluntary Aid Detachment nurses, and by nursing orderlies. The former Poor-Law probationers have been retained, and an attempt has been made to carry on their training, but this system is breaking down. It is obvious that these military hospitals cannot provide the general training in nursing the infirmaries provided. It is now becoming generally recognised that these new military hospitals will have for the time being to cease to act as training schools for nurses. The problem is not an easy one to solve without hardship to the existing probationers. In all probability the solution will be found in allowing those probationers who had eighteen months' training under the old conditions to complete their training, and in drafting the junior probationers to hospitals and infirmaries which can undertake the remainder of their training.

The general administration of these new military hospitals is in the hands of the War Office, but the payment of the staff, except the medical officers and orderlies, is made through the Guardians, who also make contracts for the supply of food, fuel, and drugs. Surgical instruments, ward equipments, structural alterations, etc., after being approved by headquarters, are usually referred to the Guardians to supply or execute. At the beginning the exact functions of the military and Poor-Law authorities were not clearly defined, and some friction arose in consequence, but the position is now understood better, and things are working much more smoothly.

The equipment of the institutions has been increased and improved in many directions, although in some instances War Office regulations have proved less elastic and generous than the provision made by the Guardians. As an example, one may adduce the supply of certain things for the use of individual patients. Cylindrical spectacles, surgical boots, and other appliances, the War Office will not sanction unless the condition requiring them has arisen as the result of active military service.

The number of beds in these hospitals varies from 500 to over a thousand. The accommodation was originally based upon that sanctioned by the Local Government Board, but the increasing demand has led to additional beds being placed in the wards; and it is now proposed, when ground is available for the purpose, to erect temporary huts. The hospitals the Poor-Law authorities have been able to place at the disposal of the War Office have been of the greatest value.

